# Does the Plague Still Threaten Us?

**DOI:** 10.1590/0037-8682-0136-2019

**Published:** 2020-03-16

**Authors:** Alzira Maria Paiva de Almeida, Marise Sobreira, Nilma Cintra Leal, Celso Tavares

**Affiliations:** 1 Fundação Oswaldo Cruz, Instituto Aggeu Magalhães, Departamento de Microbiologia, Recife, PE, Brasil.; 2 Conselho Federal de Medicina, Câmara Técnica de Infectologia, Maceió, AL, Brasil.

**Dear Editor:**

Despite decimating populations over the centuries, plague is currently an invisible zoonosis for both the state and society. Human cases of the plague have declined in the recent years. However, plague has been overlooked in medical education, and hence, most of the health professionals face difficulties in recognizing the disease symptoms. Such panorama is concerning since early identification of isolated cases may be the key to prevent the spread of an epidemic.

Brazil has several plague foci, where the agent (*Yersinia pestis*), its hosts, and vectors coexist, constituting a permanent threat to the local population and to those who visit the areas for leisure or work. These focal areas are spreading throughout the mountains of Ibiapaba and Baturité (State of Ceará) and in Chapada do Araripe (States of Ceará, Pernambuco, and Piauí), Chapada da Borborema (States of Alagoas, Paraíba, Pernambuco, and Rio Grande do Norte), Serra de Triunfo (States of Paraíba and Pernambuco), Plateau Oriental, Chapada Diamantina, Piemonte da Chapada Diamantina (State of Bahia), Vale do Rio Doce, Vale do Jequitinhonha (State of Minas Gerais), and Serra dos Orgãos (State of Rio de Janeiro)^1^.

The experience accumulated in Brazil for more than a century shows that notification and early diagnosis are essential to save the patient’s life, to identify the probable index case, and to trigger prevention activities to avoid future epidemics. Therefore, healthcare professionals must understand the regional nosology, which allows them to identify instances of plague to help confirm the diagnosis.

Plague is also an occupational hazard^2^ ^,^ ^3^. A serological survey has revealed the presence of antibodies against plague and hantavirus among healthcare professionals working in zoonosis control programs. However, they had no previous symptoms or clinical signs of plague^4^. It is advisable to populations in at-risk areas to be aware of events suggestive of plague, such as the occurrence of epizootics of rodents without apparent cause. These signs are not always perceived or valued by healthcare professionals or the general public.

Because of the lack of attention regarding plague control, it is difficult to estimate the actual plague-associated morbidity and mortality, which is aggravated because this zoonosis occurs in remote and impoverished places where the populations have limited access to healthcare services and health surveillance is practically nonexistent. Therefore, it is reasonable to speculate that cases of the disease occur, but they are not reported.

In contrast, false-positive cases result from misdiagnosis in clinical laboratories that use automated systems of microbial identification^5^. Some systems do not correctly identify *Y. pestis*, leading to a false-positive or -negative diagnosis. Because of its weak biochemical reactivity, *Y. pestis* can be confused for *Shigella, Acinetobacter, Pseudomonas,* and even other enteropathogenic and environmental *Yersinia* species^5^ ^,^ ^6^.

Therefore, in the focal areas, the general public, healthcare professionals, and health authorities should consider plague to be a real threat. Its focal condition makes it a regional nosological problem, and it can be expected that most cases will be among residents of these areas. Suspected cases outside of these focal areas should be rigorously investigated. Particular attention should be given to the events that occurred 12 days before the onset of symptoms. These events include contact with other suspected patients or animals from the focal areas and trips to plague regions of Brazil or other countries in Asia, Africa, and South and North America where the disease also occurs^7^.

On evaluating any suspected cases, it is crucial to remember that plague is a focal zoonosis. In January 2019, the press reported the occurrence of a presumed plague case in the urban area of São Gonçalo in the State of Rio de Janeiro approximately 70 km away from the plague area of Serra dos Órgãos^5^ ^,^ ^8^. The hypothesis of spillover was unlikely considering that wild rodents primarily uphold the disease, and no plague activity was recorded among them in that area.

Importing and trade of animals require special attention. These growing and profitable activities are responsible for the occurrence of plague both in endemic and non-endemic areas, putting staff and customers at risk^9^. Therefore, plague must be considered when acute febrile diseases are diagnosed in the most diverse mammal species, which exposes owners, veterinarians, and assistants to a high-risk situation^3^ ^,^ ^9^.

Human-to-human transmission is another event to consider. In a focal area in Peru in 2010, a physician and a medical student were infected with *Y. pestis* after they provided care for a patient whose initial diagnosis was community-acquired pneumonia or influenza, without the use of adequate respiratory protection. They were admitted to the intensive care unit, and the 21-year-old medical student died^10^. During an outbreak in Madagascar, 2,417 cases occurred from August to November of 2017, of which 77% presented the pneumonic form, and 81 cases occurred in health professionals^11^.

An accurate diagnosis of plague is still challenging. The predominant clinical presentation of the disease is the flea-transmitted bubonic form, which is characterized by the presence of buboes or painful adenitis. The rarer pneumonic form is transmitted from person-to-person via respiratory droplets, which causes in cough, dyspnea, chest pain, and mucus/bloody sputum. In the primary septicemic form without apparent buboes, the patients present with fever, chills, headache, generalized body aches, weakness, anorexia, hypotension, and fast/irregular pulse^12^.

Bubonic plague can be clinically mistaken for other diseases. These diseases include sexually transmitted infections, toxoplasmosis, cytomegalovirus, acute histoplasmosis, tularemia, neoplasm, ruptured hernia, rickettsioses, typhoid fever, sepsis, and other processes involving fever and lymphadenopathy. It is worth emphasizing that lymphangitis does not occur in the plague, and the buboes are extremely painful. Septicemic plague should be differentiated from bacterial septicemia and other infectious diseases of acute onset and rapid and severe course. These infections include meningococcemia, typhus, typhoid fever, malaria, dengue III and IV, and Rocky Mountain spotted fever. Pneumonic plague should be distinguished from other types of pneumonia, bronchopneumonia, and cardiopulmonary syndrome due to hantavirus. The detection of a cavitary lesion on chest radiography may suggest tuberculosis, which can be ruled out based on the natural history of the disease^13^.

Distinct sample specimens should be obtained depending on the various forms of the disease, including bubo aspirate and blood for the bubonic form and sputum for the pneumonic plague. It is essential to collect the blood for cultures in all suspected cases to determine the presence of *Y. pestis* and obtain the serum for serological tests. *Y. pestis* is a gram-negative coccobacillus of the family Enterobacteriaceae. It is categorized as a Category A bioterrorism agent requiring level 3 biosafety. The sample collection requires disposable gloves, a laboratory coat, and respiratory protection for biosafety level 3. The manipulation of biological samples for the diagnosis of plague requires a level 3 containment laboratory^12^ ^,^ ^13^.

Since the plague cannot eradicated yet, rigorous monitoring of host and vector populations would allow early detection of any activity in the wild. Such an approach triggers prompt control measures, preventing the potential spread to humans. Furthermore, it is imperative to provide continuous training to healthcare professionals in the affected areas. The education of primary care teams should focus on early detection and control. Secondary and tertiary care staff need to be aware of the clinical and epidemiological features for a precise therapeutic decision as some cases may evolve unfavorably or have clinical presentations that require special care.
